# Beyond Central Subfield Thickness: Early Multi-Slice Optical Coherence Tomography Structural Response After Faricimab Injection in Real-World Diabetic Macular Edema

**DOI:** 10.3390/jcm15166356

**Published:** 2026-08-17

**Authors:** De-Yi Liu, Shiao-Ling Wu, Ning-Yi Hsia, Peng-Tai Tien, Chun-Ju Lin, I Wang, Chun-Ting Lai, Jane-Ming Lin, Yu-Te Huang, Bing-Qi Wu, Wei-Ning Lin, Wei-Ning Ku, Ping-Ping Meng, Huan-Sheng Chen, Yi-Yu Tsai

**Affiliations:** 1 Department of Ophthalmology, China Medical University Hospital, China Medical University, Taichung 404328, Taiwan; deyil845@gmail.com (D.-Y.L.); wsl030030@gmail.com (S.-L.W.); u702054@hotmail.com (P.-T.T.); u9801310@cmu.edu.tw (I.W.); withwind037@yahoo.com.tw (C.-T.L.); d4301@seed.net.tw (J.-M.L.); tonyhuang791112@gmail.com (Y.-T.H.); jacky870914@gmail.com (B.-Q.W.); ning1128@gmail.com (W.-N.L.); yasuko721@gmail.com (W.-N.K.); c19902001@hotmail.com (P.-P.M.); yiyutsai@seed.net.tw (Y.-Y.T.); 2 Department of General Medicine, China Medical University Hospital, Taichung 404328, Taiwan; 3 School of Medicine, College of Medicine, China Medical University, Taichung 404328, Taiwan; 4 Department of Optometry, Asia University, Taichung 413305, Taiwan; 5 An-Shin Dialysis Center, Excelsior Renal Service Co., Ltd. Taiwan Branch, Taichung 436018, Taiwan

**Keywords:** diabetic macular edema, optical coherence tomography, faricimab, angiopoietin-2, biomarkers

## Abstract

**Objectives**: We sought to evaluate the early efficacy of faricimab (Vabysmo^®^) in treating diabetic macular edema (DME) and to explore the predictive value of multi-slice optical coherence tomography (OCT) biomarkers for anatomical and visual outcomes. **Methods**: In this retrospective cohort study, 26 anti-VEGF-naive DME patients (36 eyes) treated with intravitreal faricimab were analyzed from baseline through 6 months of follow-up. Best-corrected visual acuity (BCVA) was recorded, while central subfield thickness (CST) and various OCT biomarkers were evaluated using multi-slice OCT quantitative analysis. Logistic and linear regression models were utilized to examine predictive factors, and scatter plots were employed to assess the correlation between anatomical improvement and functional visual gain. **Results**: Changes in CST and BCVA, along with the evolution and predictive power of OCT biomarkers—including vitreomacular interface (VMI), epiretinal membrane (ERM), disorganization of the retinal inner layers (DRIL), intraretinal cysts (IRCs), hyperreflective foci (HRF), hard exudates (HEs), large outer-nuclear-layer cavities (LONLCs), ellipsoid zone disruption (EZD), and subretinal fluid (SRF)—were assessed. Post-treatment CST demonstrated rapid and significant reduction, decreasing from 377.7 μm (95% CI: 347.0–408.4) at baseline to 312.8 μm (95% CI: 287.5–338.2; *p* < 0.0001) at month 3 and 291.5 μm (95% CI: 275.6–307.4; *p* < 0.0001) at month 6, with an approximately 48 μm reduction post-first injection. Overall intraocular pressure (IOP) and BCVA showed no statistically significant improvement. Baseline analysis indicated that EZD was significantly associated with older age (*p* = 0.029), worse initial BCVA (*p* = 0.005), and thicker CST (*p* = 0.002). After adjusting for initial CST in the linear regression model, we identified the presence of baseline HEs as the sole independent predictor of substantial anatomical improvement (B = 45.9, *p* = 0.002). **Conclusions**: Faricimab demonstrated rapid and significant early anatomical improvements. Baseline HEs independently predicted the extent of CST reduction, potentially reflecting the greater fluid burden of eyes with more severe barrier breakdown. Baseline EZD was observed exclusively among eyes that did not achieve complete anatomical remission, although the small number of EZD-positive eyes (*n* = 7) precludes firm conclusions.

## 1. Introduction

Diabetic macular edema (DME) is characterized by fluid accumulation in the macula resulting from breakdown of the inner blood–retinal barrier, representing the most common cause of visual impairment in patients with diabetes, affecting approximately 3.8–5.5% of adults with diabetes—more than 20 million people worldwide [1,2]. At the molecular level, overexpression of vascular endothelial growth factor (VEGF) compromises tight-junction integrity, leading to increased vascular permeability and macular edema [3]. Beyond vascular endothelial growth factor, chronic low-grade inflammation and dysregulation of the Angiopoietin/Tie-2 pathway further contribute to pericyte loss, vessel destabilization, and sustained vascular inflammation, together leading to structural retinal damage [3,4]. Left untreated, DME is a leading cause of central vision loss and blindness in working-age adults, significantly impairing independence and quality of life [1].

Intravitreal anti-VEGF therapy has been recommended as first-line treatment for center-involved DME (CI-DME) since 2010 [5,6]. Despite these advances, 30–50% of patients fail to achieve optimal outcomes with anti-VEGF monotherapy [7,8,9,10,11], suggesting that VEGF-independent pathways play an important role in disease persistence. Faricimab (Vabysmo^®^) addresses this limitation, serving as the first bispecific antibody designed to simultaneously neutralize both VEGF-A and Angiopoietin-2 (Ang-2), stabilizing retinal vasculature through restoration of Tie-2 signaling while suppressing VEGF-driven permeability [12]. Results from the landmark Phase 3 YOSEMITE and RHINE clinical trials indicated that faricimab achieved functional visual improvements comparable to aflibercept, even when treatment intervals were extended up to 16 weeks [6,12].

As the primary imaging modality for diagnosing and tracking DME, optical coherence tomography (OCT) facilitates the objective measurement of various morphological features. These clinically relevant parameters include epiretinal membrane (ERM), subretinal fluid (SRF), intraretinal cysts (IRCs), hard exudates (HEs), hyperreflective foci (HRF), ellipsoid zone disruption (EZD), and disorganization of the retinal inner layers (DRIL) [13,14]. DRIL and EZD are strong independent predictors of poor visual recovery following anti-VEGF therapy [14], while HRF and ERM have been associated with treatment resistance and may help guide selection between anti-VEGF and steroid-based therapies [15,16,17]. Collectively, these biomarkers show clinically meaningful associations with disease severity, inflammatory activity, and differential treatment responses [13,14,15,16], underscoring the potential of a multi-biomarker OCT approach to individualize DME management.

Although baseline OCT biomarkers have been evaluated in the context of conventional anti-VEGF therapy, their predictive value specifically for faricimab remains insufficiently characterized. Given faricimab’s unique mechanism, structural biomarkers reflecting vascular integrity and inflammatory activity may carry different, or enhanced, predictive significance compared to anti-VEGF monotherapy. Therefore, this study introduces a multi-slice OCT biomarker framework that captures spatial heterogeneity beyond central subfield thickness. The present study seeks to determine the initial therapeutic response to faricimab in patients with treatment-naïve DME. Furthermore, we explore how baseline OCT features predict subsequent structural and functional results, ultimately aiming to facilitate personalized, biomarker-driven treatment strategies for this disease.

## 2. Materials and Methods

### 2.1. Study Design and Patient Selection

We conducted a retrospective, observational review of clinical records from patients managed at China Medical University Hospital (CMUH), a tertiary care center in Taiwan, between March 2024 and December 2025. This research was performed in compliance with the Declaration of Helsinki guidelines and was granted ethical clearance by the CMUH Institutional Review Board (approval number: CMUH115-REC1-089). IRB approval was obtained on April 18, 2026, strictly prior to any data extraction or analysis of these historical records. Due to the retrospective nature of the study design, the requirement for informed consent was waived by the review board.

Patients were eligible for inclusion if they met the following criteria: (1) age 18 years or older; (2) an established diagnosis of type 1 or type 2 diabetes mellitus; (3) macular edema secondary to either proliferative or non-proliferative diabetic retinopathy, confirmed by fluorescein angiography showing macular leakage; (4) completely naïve to previous anti-VEGF therapies; (5) a baseline Snellen best-corrected visual acuity (BCVA) ranging from 20/400 to 20/40; (6) an initial central subfield thickness (CST) exceeding 300 μm on OCT; (7) treated solely with 6.0 mg intravitreal faricimab (IVF) injections during the study; and (8) availability of complete BCVA and OCT records at baseline, after the initial injection, and at the 3- and 6-month follow-up visits. For individuals with bilateral DME undergoing IVF treatment in both eyes, each eye was included in the analysis as a separate entity.

Criteria for exclusion consisted of the following: (1) coexisting retinal or macular pathologies, aside from diabetic retinopathy, capable of inducing macular edema; (2) any prior vitreoretinal surgical intervention; (3) poorly controlled glaucoma; (4) systemic or ocular comorbidities that could independently affect visual acuity; (5) patients with PDR who required or received additional macular laser photocoagulation during the 6-month study period; and (6) patients who received dexamethasone intravitreal implant before or during the 6-month study period. Furthermore, OCT scans deemed of insufficient or poor image quality by the masked evaluators were excluded from the morphological analysis. Comprehensive medical chart reviews were conducted to extract baseline demographics, type and severity of retinopathy, and relevant systemic parameters. Clinical parameters, specifically CST, intraocular pressure (IOP), and BCVA, were documented at four critical timepoints: baseline, following the first IVF dose, and at the 3- and 6-month visits. The treatment regimen began with a loading series of IVF injections, subsequently transitioning to a personalized dosing schedule. This maintenance phase employed established pro-re-nata (PRN) or treat-and-extend (T&E) strategies, adjusting intervals according to the patient’s anatomical (CST) and functional (BCVA) stability. The treatment interval was extended by 4 weeks if the treated eye achieved functional and anatomical stability, defined as a stable BCVA, a CST of less than 300 μm, and the complete resolution of intraretinal or subretinal fluid. Conversely, if there was documented visual deterioration or recurrent macular edema (CST ≥ 300 μm or recurrent fluid), the injection interval was shortened.

### 2.2. Imaging and Data Acquisition

**Figure 1:**
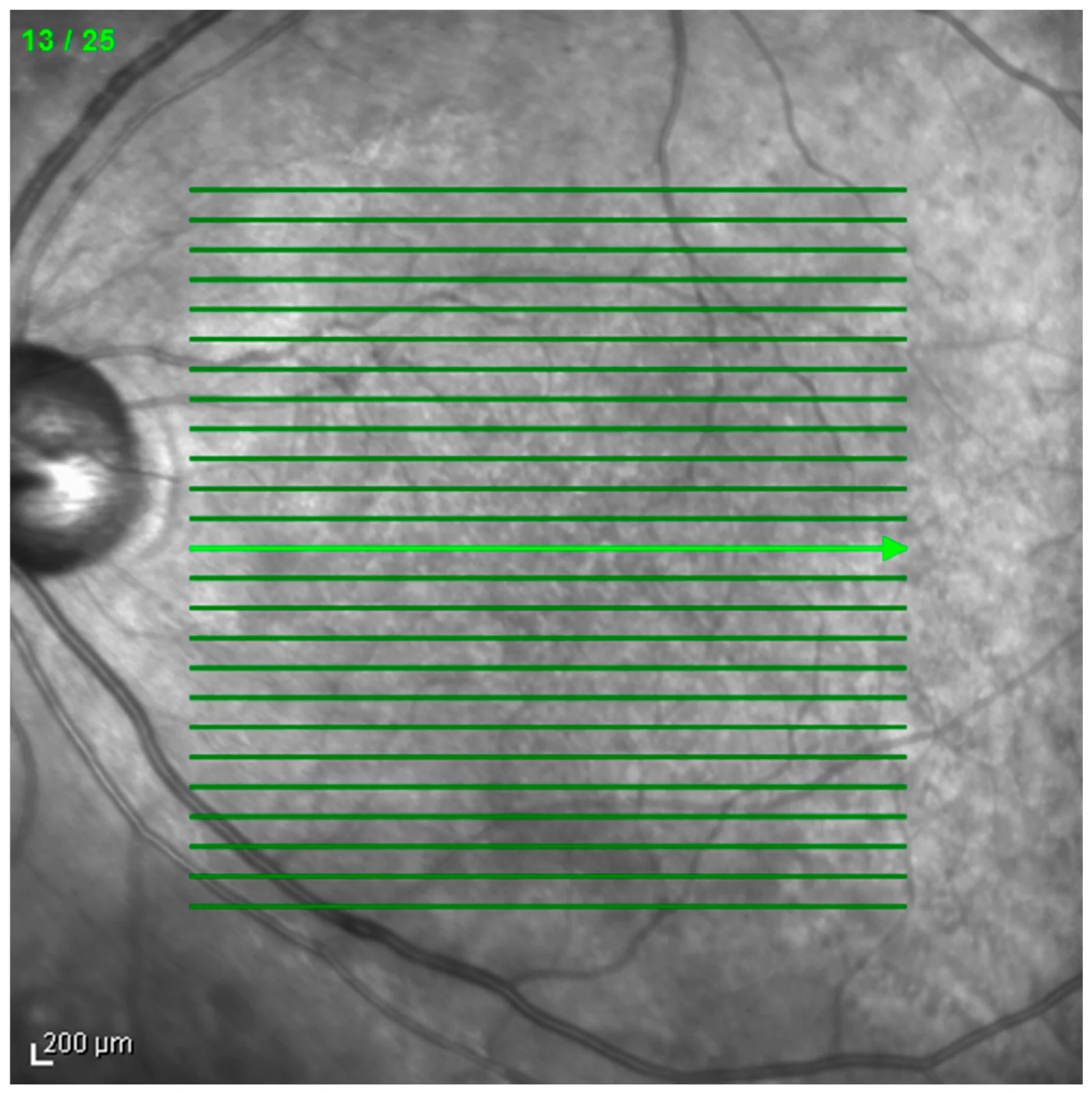
Multi-slice SD-OCT consisting of 25 horizontal B-scans over a 6 × 6 mm area. Standardized macular volume scans centered on the fovea, consisting of 25 horizontal B-scans covering a 6 × 6 mm area, acquired using SD-OCT (Heidelberg Spectralis). Multi-slice analysis enabled comprehensive qualitative and quantitative evaluation of OCT biomarkers at baseline and throughout faricimab treatment. Abbreviations: OCT, optical coherence tomography; SD-OCT, spectral-domain optical coherence tomography.

Imaging was consistently performed using a Spectral-Domain OCT (SD-OCT) system (Heidelberg Spectralis, Heidelberg, Germany). For every enrolled eye, a standardized fovea-centered macular volume protocol was executed, capturing a 6 × 6 mm grid comprised of 25 horizontal B-scans (Figure 1). These multi-slice SD-OCT scans underwent detailed qualitative and quantitative analysis at four predefined intervals: prior to treatment, after the initial injection, and at the 3- and 6-month follow-up marks.

We systematically assessed the presence of nine specific OCT morphological biomarkers of interest: (1) VMI; (2) ERM; (3) DRIL; (4) IRCs; (5) HRF; (6) HEs; (7) LONLCs; (8) EZD; and (9) SRF.

The morphological definitions for each OCT biomarker were established as follows: HRF were defined as discrete, well-circumscribed, dot-like lesions within the neurosensory retina presenting reflectivity comparable to that of the retinal pigment epithelium (RPE) band. SRF was identified as continuous hyporeflective spaces accumulating between the neurosensory retina and the RPE. IRCs were defined as round or oval hyporeflective spaces located within the boundaries of the neurosensory retina. LONLCs were identified as prominent hyporeflective cystic areas measuring greater than 100 μm in diameter, exclusively situated within the outer nuclear layer. EZD was recognized as any loss of continuity or focal attenuation within the highly reflective ellipsoid zone band. DRIL was characterized by the inability to clearly delineate the boundaries between the ganglion cell-inner plexiform layer complex, inner nuclear layer, and outer plexiform layer. HEs were identified as hyperreflective foci within the retina that cast a prominent, characteristic posterior optical shadowing effect. ERM was characterized by the presence of a hyperreflective fibrocellular tissue adhering to the internal retinal surface. Furthermore, anomalies of the VMI were categorized as any architectural disturbances at the vitreoretinal boundary, which included vitreomacular traction, vitreomacular adhesion, and posterior vitreous detachment.

To maintain unbiased assessments, all OCT scans were manually graded by two independent, masked reviewers (DYL and SLW). During the entirety of the imaging evaluation, these graders were completely blinded to the subjects’ systemic health profiles, visual acuity results, and overall clinical trajectories. In the event of a discrepancy between the two primary evaluators, two senior retina specialists (CJL and NYH) were consulted to adjudicate and establish the final consensus grade. Interobserver agreement for the presence or absence of qualitative OCT biomarkers was assessed using Cohen’s κ coefficient. Computer-aided quantitative analyses of OCT images via built-in software were utilized to determine objective anatomical metrics such as CST.

### 2.3. Statistical Analysis

We utilized IBM SPSS Statistics software, version 25.0 (IBM Corp., Armonk, NY, USA), to conduct all statistical computations. Additionally, GraphPad Prism version 7.0 (GraphPad Software, San Diego, CA, USA) was employed to construct the data visualizations.

When both eyes of a patient met the inclusion criteria, data from both eyes were included in the analyses. Eyes were analyzed as independent observational units regardless of whether one or both eyes were contributed by an individual patient, and no statistical adjustment was made for within-patient correlation. Continuous variables were reported as means accompanied by their standard deviations (SD), whereas categorical parameters were described utilizing raw counts and their corresponding percentages. Baseline clinical parameters and their associations with biomarker positivity were analyzed using Fisher’s exact test or Chi-square test. For these categorical analyses, continuous baseline variables were dichotomized using clinically relevant cut-offs: age (≤65 vs. >65 years), initial BCVA (Logarithm of the Minimum Angle of Resolution [LogMAR] ≤ 0.4 vs. >0.4), and initial CST (≤350 μm vs. >350 μm). Longitudinal changes in continuous variables (e.g., CST, BCVA) across the predefined time points were analyzed using one-way repeated measures ANOVA or paired t-tests. Changes in the prevalence of categorical OCT biomarkers between baseline and the end of the study within specific outcome subgroups were evaluated using McNemar’s test.

To identify predictive factors for treatment outcomes, two distinct modeling approaches were employed. Univariate and multivariate logistic regression models were utilized to evaluate the predictive value of baseline OCT biomarkers for categorical visual and anatomical outcomes. For the anatomical outcomes, eyes were classified into two distinct categorical metrics: (1) the magnitude of CST improvement (stratified as reduction < 50 μm vs. ≥50 μm), and (2) the final anatomical response. Based on the strict criteria established by the DRCR.net Protocol T, ‘good responders’ were defined as eyes achieving anatomical remission (a central thickness of <320 µm for men and <305 µm for women on Heidelberg Spectralis OCT) with complete resolution of fluid, whereas ‘suboptimal responders’ were defined as those failing to meet these sex-specific thresholds [18]. Because of the limited sample size, OCT biomarkers were evaluated individually rather than entered simultaneously into a single multivariable model. Separate adjusted models were constructed for each OCT biomarker, with baseline CST included as a prespecified covariate. Similarly, separate general linear models (GLMs) were constructed for each OCT biomarker, adjusting for baseline CST, to determine whether each biomarker independently predicted the continuous extent of CST reduction (ΔCST) following treatment.

## 3. Results

### 3.1. Baseline Demographics and Clinical Characteristics

A total of 26 patients contributed 36 eyes with DME treated with faricimab, and the eye was the unit of analysis throughout the study, aside from patients’ age, sex distribution, and HbA1c percentage. The study population had an average age of 57.8 ± 13.4 years and included 53.8% female patients, with a mean baseline HbA1c of 7.9 ± 1.7%. Initial clinical assessments revealed an average CST of 377.7 ± 90.7 μm alongside a mean BCVA of 0.5 ± 0.4 LogMAR (Table 1). Regarding the morphological evaluation, the qualitative grading of OCT biomarkers demonstrated high interobserver reproducibility between the two masked evaluators, with an overall agreement rate of 94.2% and a Cohen’s κ coefficient of 0.86. Disagreements in the remaining cases were successfully resolved through senior adjudication. Further analysis of the associations between baseline OCT biomarkers and clinical parameters (Table S1) revealed that the incidence of EZD was significantly associated with older age (*p* = 0.029), worse initial BCVA (LogMAR > 0.4, *p* = 0.005), and thicker initial CST (>350 μm, *p* = 0.002). The presence of SRF was significantly associated with worse initial BCVA (*p* = 0.016). Moreover, eyes presenting with an initial CST > 350 μm exhibited a significantly higher prevalence of DRIL and LONLCs (*p* < 0.001 and *p* = 0.02, respectively).

### 3.2. Anatomical and Functional Outcomes

**Figure 2:**
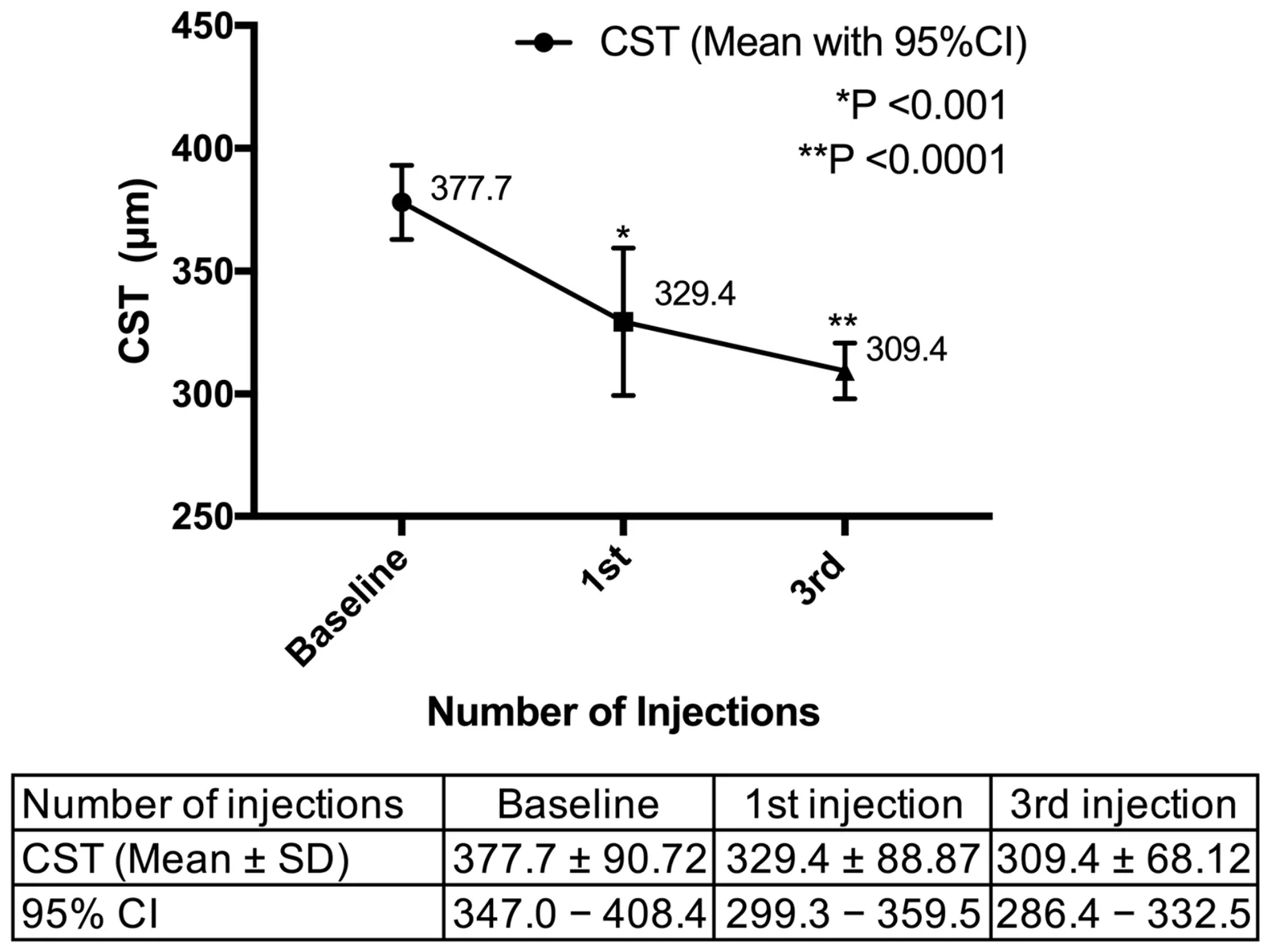
Average CST in all patients by number of injections. Mean CST at baseline, post-first injection, and post-third injection. Note: ‘post-third injection’ refers to the evaluation following the third dose, which may vary chronologically due to individualized PRN/T&E regimens, and is distinct from the fixed 3-month chronological follow-up presented in Figure 3. Error bars represent 95% confidence intervals. * *p* < 0.001; ** *p* < 0.0001. Abbreviations: CI, confidence interval; CST, central subfield thickness; SD, standard deviation.

**Figure 3:**
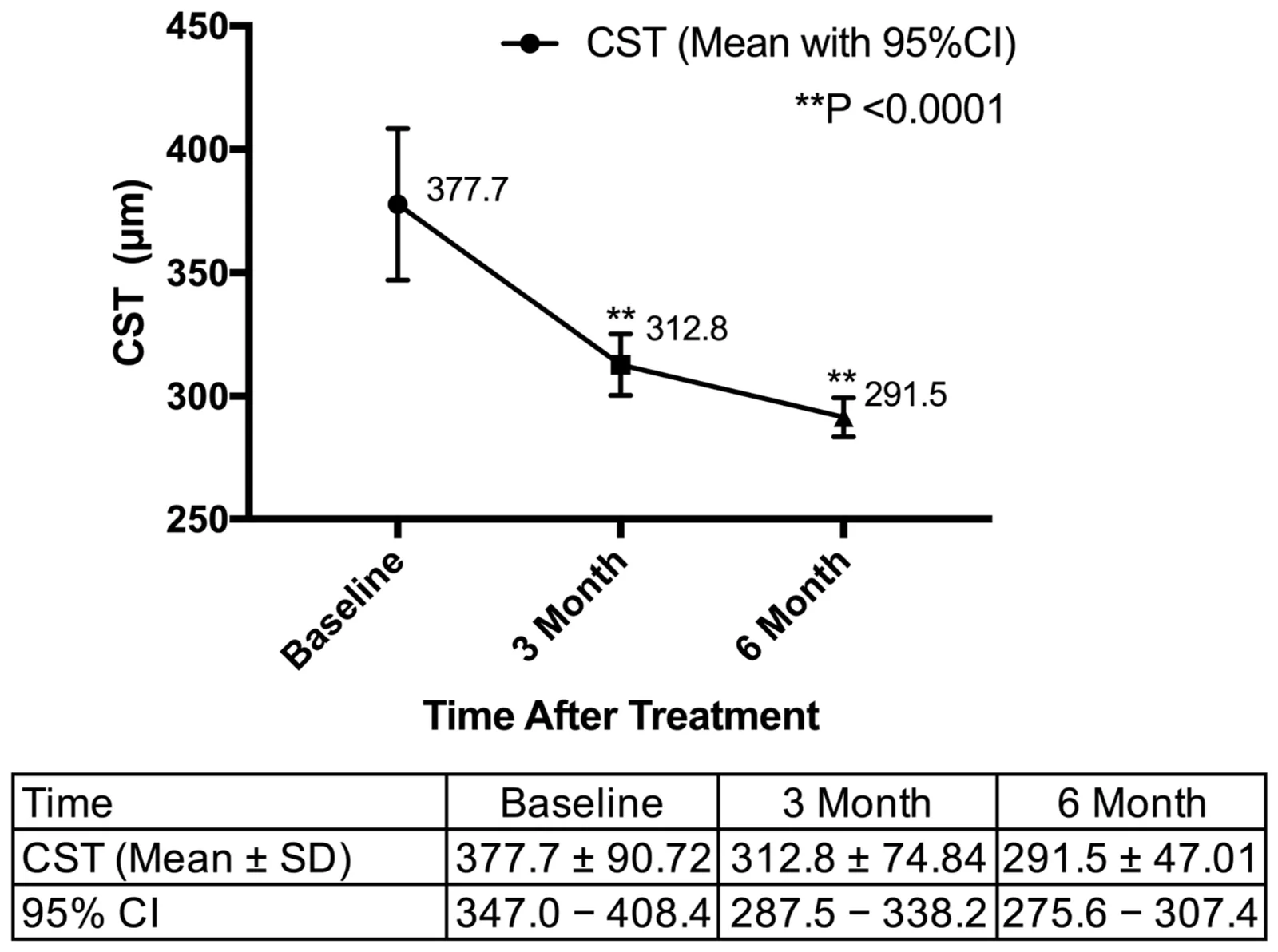
Average CST in all patients by treatment duration. Mean CST at baseline, 3-month, and 6-month follow-up visits. Error bars represent 95% confidence intervals. ** *p* < 0.0001 Abbreviations: CI, confidence interval; CST, central subfield thickness; SD, standard deviation.

Following faricimab treatment, the eyes exhibited robust and expedited anatomical resolution. The mean CST decreased significantly from 377.7 μm (95% confidence interval [CI]: 347.0–408.4) at baseline to 329.4 μm (95% CI: 299.3–359.5) after the first injection (*p* < 0.001) (Figure 2). This structural improvement was sustained at the 3-month and 6-month follow-ups, with mean CSTs further decreasing to 312.8 μm (95% CI: 287.5–338.2, *p* < 0.0001) and 291.5 μm (95% CI: 275.6–307.4, *p* < 0.0001), respectively (Figure 3). Specifically, the mean within-eye change in CST from baseline to month 3 was −48.6 μm (95% CI: −71.8 to −25.5 μm), and to month 6 was −86.2 μm (95% CI: −113.5 to −58.9 μm). Despite significant and rapid anatomical restoration, changes in overall BCVA and IOP did not reach statistical significance throughout the 6-month observation period (Figures S1 and S2).

### 3.3. Evolution of OCT Biomarkers and Structural Fixation

The longitudinal evolution of OCT biomarkers pre- and post-treatment is detailed in Table S2. Across the entire cohort, the prevalence of IRCs, DRIL, and LONLCs decreased significantly following treatment. Notably, no newly incident cases of ERM were observed during the 6-month follow-up; all documented ERMs were pre-existing at baseline.

Subgroup analysis of the suboptimal responders unveiled a critical pathological phenomenon. Although biomarkers such as SRF, DRIL, and LONLCs showed improvement in this group, the proportion of IRCs remained exceptionally high, persisting at 91.7% from baseline to the study conclusion (*p* = 1.0). Compared to other biomarkers, IRCs demonstrated recalcitrant structural fixation in these refractory cases, representing a profound pathological limitation to complete anatomical restoration.

### 3.4. Predictive Factors for Treatment Outcomes

To identify potential predictors of treatment response, baseline characteristics were evaluated against the magnitude of CST improvement (<50 μm vs. ≥50 μm) (Table 2) and the final anatomical outcomes (good vs. suboptimal responders) (Table 3). The analysis revealed that, although OCT biomarkers did not show statistically significant differences in the multivariate models after adjusting for baseline CST, baseline EZD was present in 0% of good responders compared with 29.2% of suboptimal responders (Table 3), and a comparable pattern was observed for the magnitude of CST improvement (Table 2). Because of the zero cell, odds ratios were not estimable for either comparison.

**Figure 4:**
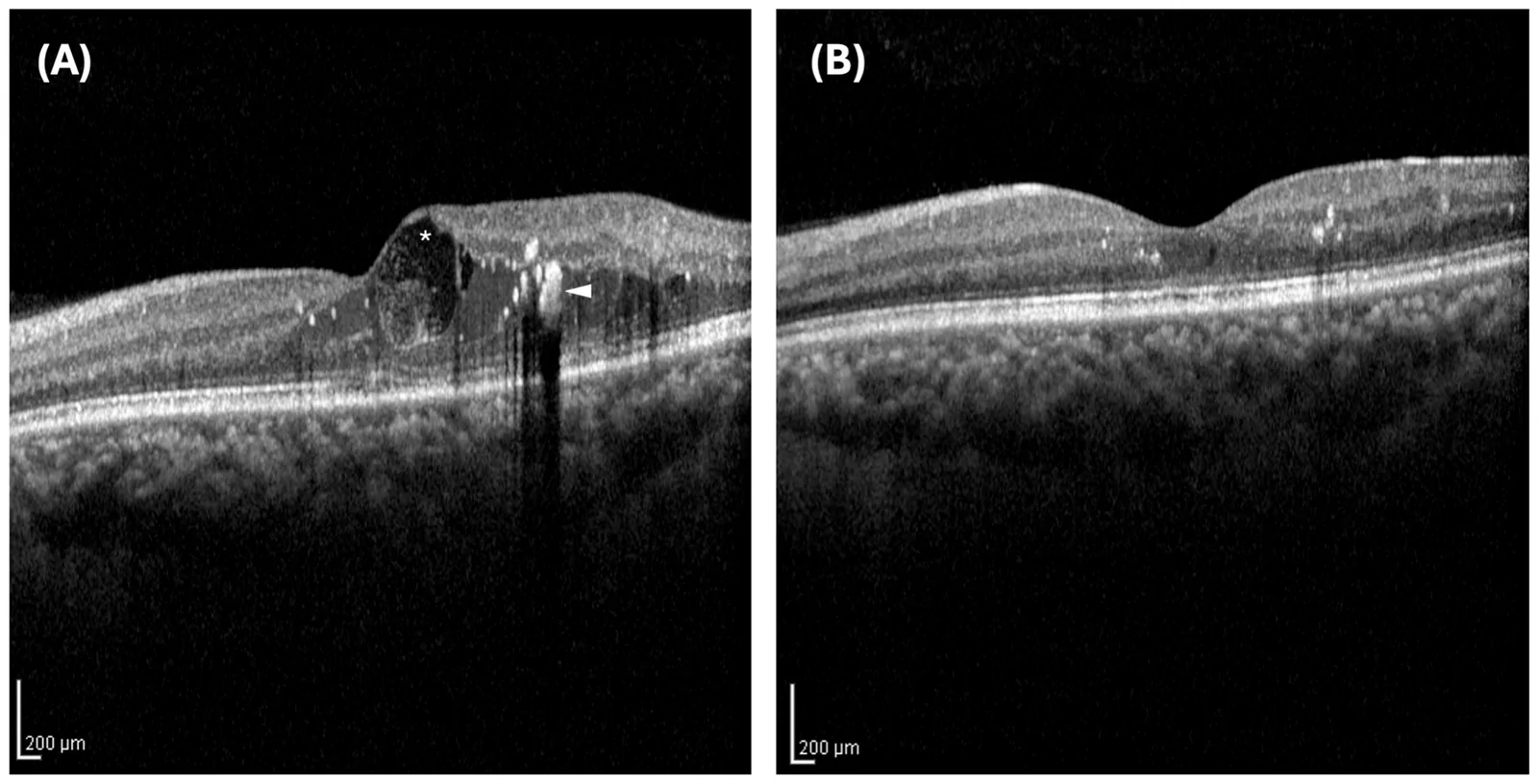
Representative multi-slice OCT images of a DME eye before and 6 months after faricimab treatment. Representative structural and functional improvements in a DME patient treated with faricimab. (**A**) Baseline OCT scan of a 49-year-old male patient demonstrates significant macular edema with prominent IRCs (asterisks) and HEs (arrowhead) (CST: 354 μm, LogMAR: 0.15). (**B**) At 6 months following faricimab therapy, the OCT scan reveals marked anatomical resolution and functional visual recovery (CST: 251 μm, LogMAR: 0), characterized by the near-complete resolution of both IRCs and HEs. Both panels represent the exact same registered B-scan location. Note: While the baseline LogMAR of this specific patient is better than the cohort mean, this case was selectively chosen to best illustrate the pronounced structural clearance of prominent baseline HEs following faricimab therapy. Abbreviations: CST, central subfield thickness; DME, diabetic macular edema; HEs, hard exudates; IRCs, intraretinal cysts; LogMAR, Logarithm of the Minimum Angle of Resolution; OCT, optical coherence tomography.

Furthermore, in a general linear model evaluating the magnitude of Δ CST, after adjusting for baseline CST, the presence of HEs emerged as the sole independent predictor for significant CST reduction (Unstandardized Coeff. B = 45.98, *p* = 0.002) (Table 4). A representative case illustrating this robust anatomical response in a patient presenting with prominent baseline HEs is shown in Figure 4. While HEs did not reach statistical significance in multivariate logistic regression, they remained a significant independent predictor in the GLM model. This indicates that eyes with baseline HEs possessed a greater potential for substantial physical thickness reduction following faricimab therapy.

## 4. Discussion

This retrospective study evaluated the early efficacy of the bispecific antibody faricimab in a real-world DME cohort and investigated the predictive value of OCT biomarkers. Our main findings demonstrate that faricimab achieves rapid and significant early anatomical resolution. Notably, baseline HEs served as an independent predictor for the magnitude of CST reduction. Additionally, no new-onset ERM was observed throughout the follow-up period.

### 4.1. Early Anatomical Response and the Anatomical–Functional Disconnect

In our cohort, faricimab exhibited significant fluid clearance immediately after the first injection, achieving a mean CST reduction of approximately 48 μm that was sustained through 6 months. This rapid early response closely mirrors recent real-world data reported by Kusuhara et al., who observed a comparable initial CST reduction (approximately 54 μm) within the first month of faricimab treatment [19]. The parallel magnitude of early fluid clearance across independent real-world cohorts highlights the consistent early efficacy of faricimab. While the reductions of our real-world study appear numerically smaller than those reported in the pivotal phase 3 YOSEMITE and RHINE trials [12], this discrepancy might primarily be attributable to a ceiling effect. Our patients presented with a relatively lower baseline CST (377.7 μm), leaving less physical space for thickness reduction.

However, achieving a near-normal retinal thickness (329.4 μm) after a single dose still supports the potent vascular stabilizing and anti-leakage effects driven by its unique bispecific mechanism. Faricimab is designed to simultaneously and independently bind and neutralize both Ang-2 and VEGF-A [12]. While the suppression of VEGF-A halts acute endothelial hyperpermeability, the concurrent inhibition of Ang-2 plays a complementary and synergistic role by restoring long-term vascular stability. This dual-pathway blockade effectively addresses the multifactorial pathogenesis of DME, explaining the rapid anatomical resolution observed in our real-world cohort.

Despite significant CST reduction, mean BCVA did not improve significantly over 6 months. This dissociation likely reflects a ceiling effect from relatively preserved baseline acuity (mean 0.5 LogMAR; inclusion capped at 20/40), limited power in a 36-eye cohort, and the fundamental distinction between structural resolution and functional recovery—the latter depending on the integrity of the photoreceptor–RPE complex and the inner retinal neurons that may already be compromised at treatment onset. Consistent with this, baseline EZD was associated with worse presenting BCVA (*p* = 0.005), and DRIL, an established predictor of poor visual recovery, was present in 55.6% of eyes at baseline. This anatomical–functional disconnect is well described across anti-VEGF agents and reinforces the rationale for our biomarker framework: structural improvement alone is an incomplete measure of benefit.

### 4.2. HEs, a Marker of Leakage Severity, and Observations on ERM

Baseline HEs emerged as the only independent predictor of the magnitude of CST reduction in our GLM. Pathologically, HEs represent extravasated lipoprotein deposits that accumulate when plasma constituents cross a compromised blood–retinal barrier, subsequently requiring macrophage-mediated phagocytosis [20,21]. Eyes with prominent baseline HEs had more severe barrier breakdown, greater accumulated fluid, and correspondingly greater scope for measurable reduction—despite adjustment for baseline CST, residual confounding by disease severity is likely. A contributory pharmacological effect is nonetheless plausible: the pathological upregulation of Ang-2 in the diabetic macular microenvironment destabilizes the endothelium, promotes pericyte loss, and amplifies leukocyte adhesion and low-grade inflammation [4,22], and faricimab’s Ang-2 blockade may restore endothelial stability and reduce leakage by promoting pericyte recruitment while exerting anti-inflammatory effects by decreasing vascular sensitivity to VEGF [22,23]. Eyes with severe barrier compromise, of which an HE is a visible marker, may therefore be those in which dual-pathway inhibition has the greatest anatomical impact. However, our single-arm design provides no evidence that this effect is specific to faricimab.

Furthermore, over the 6-month follow-up, we observed zero cases of new-onset ERM. ERM formation most commonly follows posterior vitreous detachment, which produces dehiscence of the internal limiting membrane; glial cells migrate through these defects onto the retinal surface and, after interacting with hyalocytes, transdifferentiate into fibroblast-like cells. In secondary ERM such as that arising in diabetic retinopathy, inflammation additionally drives cell infiltration and migration, promoting fibrocellular growth [24]. Faricimab may plausibly act on this second arm: whereas traditional anti-VEGF monotherapy has been proposed to shift the intraocular cytokine balance toward an angio-fibrotic profile, Tie2 activation through Ang-2 blockade may attenuate early fibroblast activation and aberrant fibrotic remodeling [25,26,27,28]. Our observation is directionally consistent with a recent post-hoc analysis of YOSEMITE and RHINE, which reported a lower risk of ERM formation with faricimab than aflibercept [29]. This observation should nonetheless be regarded as exploratory and hypothesis-generating rather than as evidence of a faricimab-specific protective effect. Fourteen eyes (38.9%) already had ERM at baseline, leaving only 22 eyes at risk of incident ERM. Our study included no comparator arm and was not designed or powered to assess ERM development, and over 6 months the expected number of incident cases in a cohort of this size is low.

### 4.3. Structural Fixation and Baseline Outer Retinal Integrity

Despite the excellent anti-leakage efficacy of faricimab, complete anatomical resolution remains constrained by pre-existing structural damage. Our data raise the possibility that baseline EZ integrity is associated with the likelihood of a favorable anatomical response, though the small number of EZD-positive eyes means this must be interpreted as a preliminary signal rather than an established threshold. This corroborates previous findings that the integrity of the outer retina and RPE is essential for the retinal pump to effectively eliminate interstitial fluid [30,31,32]. The EZ corresponds to the mitochondria-rich inner segments of the photoreceptors, which are metabolically and functionally coupled with the underlying RPE [33]. The RPE functions as the primary active pump driving the outward clearance of interstitial fluid across the outer-retinal barrier [34,35]. We postulate that while faricimab robustly seals the inner blood–retinal barrier to halt acute vascular leakage, the clearance of pre-existing fluid relies heavily on the functional integrity of the outer retinal pump [32]. Baseline EZD may signify chronic ischemic damage and metabolic compromise of the photoreceptor-RPE complex, such that the active transport mechanisms required for maximal fluid extrusion are impaired. This may offer one plausible explanation for why eyes with baseline EZD in our cohort did not reach complete anatomical remission, and the observation requires prospective confirmation.

Moreover, recalcitrant structural fixation was observed in the suboptimal responder group, where 91.7% of eyes retained IRCs throughout the study. Experimental and histologic data indicate that severe or prolonged Müller cell injury leads to reactive gliosis and glial scar formation, with Müller cells filling injury gaps and contributing to persistent intraretinal cavities and tissue remodeling [36,37,38]. Such glial scars represent long-lasting structural changes and are unlikely to fully reverse even when vascular permeability is successfully normalized [39,40].

### 4.4. Strengths and Limitations

Our research possesses distinct advantages. First, the strictly anti-VEGF-naïve enrollment eliminates confounding from prior treatment, providing a clean baseline for evaluating faricimab’s early efficacy. Second, all OCT images were acquired using Heidelberg Spectralis SD-OCT with standardized 25 B-scan macular volume protocol over a 6 × 6 mm area, ensuring high-resolution and reproducible structural measurements. Third, we simultaneously evaluated nine OCT biomarkers—HRF, SRF, IRCs, LONLCs, EZD, DRIL, HEs, ERM, and VMI—representing one of the most comprehensive multi-biomarker analyses reported in the faricimab DME literature to date. Fourth, dual masked grading with senior adjudication minimized subjective bias in biomarker assessment. Finally, our cohort’s relatively lower mean baseline CST (377.7 μm) compared to pivotal trials such as YOSEMITE and RHINE (460–490 μm) [12] may better reflect patients encountered in routine clinical practice, where treatment is often initiated at earlier stages of disease before severe edema has accumulated.

Nevertheless, several limitations must be acknowledged. The single-center, retrospective framework of the analysis inherently carries a risk of selection bias and restricts the ability to extrapolate these findings to a wider, more diverse demographic. The relatively small sample size of 26 patients (36 eyes) reduces statistical power, may limit detection of associations with smaller effect sizes, and introduces a risk of model overfitting in multivariate analyses. Furthermore, although some patients contributed both eyes, the analyses treated each eye as an independent observation without explicitly accounting for within-patient correlation. Given the limited number of patients contributing bilateral data, the information available for estimating within-patient correlation was limited. Consequently, this approach may have underestimated standard errors and should be considered when interpreting the statistical significance of the findings. Future studies with larger cohorts should employ statistical methods, such as generalized estimating equations or mixed-effects models, to account for inter-eye correlation. These limitations may also have contributed to the discrepancies observed between regression models, suggesting potential instability in predictor identification and warranting cautious interpretation. The absence of a comparator arm precludes direct comparison with other anti-VEGF agents. Additionally, the 6-month follow-up period may be insufficient to capture longer-term anatomical and functional trajectories, particularly regarding the durability of faricimab’s extended dosing intervals; as DME arises in the context of a progressive systemic disease, this interval may also be too short to reflect longer-term disease control. We also lack data on near visual function, patient-reported outcomes, and indocyanine green angiography for evaluating telangiectatic capillaries, which are associated with circinate hard exudates and reduced anti-VEGF responsiveness—none of which were part of routine care in this cohort. The variable number of injections across patients (mean 4.2 ± 1.1), resulting from individualized PRN or T&E protocols, acts as a potential confounder and introduces heterogeneity when comparing longitudinal outcomes at fixed timepoints. Finally, as a retrospective study, unmeasured confounders, including systemic diabetic control trajectory, serum lipid profiles and other comorbidities, cannot be fully excluded from influencing the results.

## 5. Conclusions

Faricimab provided rapid and sustained anatomical improvements in anti-VEGF-naïve DME patients, without corresponding functional gain observed over 6 months. Baseline HEs may identify eyes with greater anatomical responsiveness, potentially reflecting the more severe barrier breakdown and greater fluid burden of such eyes. Baseline EZD was observed only among eyes that did not achieve complete anatomical remission; given the small number of such eyes, this represents a hypothesis-generating observation requiring confirmation. Our study introduces a multi-slice OCT biomarker framework that captures spatial heterogeneity beyond central subfield thickness. These findings highlight the value of biomarker-guided interpretation in bridging the gap between anatomical success and functional outcomes and supporting future individualized treatment strategies in DME.

## Figures and Tables

**Table 1:** Baseline demographics of all study patients.

Variables	All Patients
Patients, no.	26
- Age (years), Mean ± SD	57.8 ± 13.4
- Sex, female/total (%)	14/26 (53.8)
- HbA1c (%) *, Mean ± SD	7.9 ± 1.7
Eyes, no.	36
- Laterality, OD/total (%)	16/36 (44.4)
- Numbers of IVF injection, Mean ± SD	4.2 ± 1.1
- Lens, pseudophakic/total (%)	10/36 (27.8)
- Initial CST (μm), Mean ± SD	377.7 ± 90.7
- Initial LogMAR, Mean ± SD	0.5 ± 0.4
- Initial IOP (mmHg), Mean ± SD	17.3 ± 2.7
DR severity, *n* (%)	
- Mild NPDR	5 (13.9)
- Moderate NPDR	8 (22.2)
- Severe NPDR	5 (13.9)
- Severe NPDR s/p PRP	5 (13.9)
- PDR	2 (5.6)
- PDR s/p PRP	11 (30.6)

Abbreviations: CST, central subfield thickness; DR, diabetic retinopathy; IOP, intraocular pressure; IVF, intravitreal faricimab; LogMAR, logarithm of the minimum angle of resolution; NPDR, non-proliferative diabetic retinopathy; OD, right eye; PDR, proliferative diabetic retinopathy; PRP, panretinal photocoagulation; SD, standard deviation. * Available cases only.

**Table 2:** OCT biomarkers as predictors for CST improvement.

Parameter	Final CST Improved <50 μm (*n* = 16)	Final CST Improved ≥50 μm (*n* = 20)	Univariate			Multivariate		
			Crude OR	95% CI	*p* Value	Adjusted OR *	95% CI	*p* Value
CST, Mean ± SD	317.1 ± 38.2	426.2 ± 20.5	1.027	1.008, 1.047	0.006	—	—	—
SRF, *n* (%)	1 (6.25%)	5 (25.0%)	5	0.520, 48.068	0.163	3.254	0.231, 45.801	0.382
IRC, *n* (%)	10 (62.5%)	19 (95.0%)	11.4	1.200, 108.293	0.034	4.001	0.353, 45.353	0.263
ERM, *n* (%)	7 (43.8%)	7 (35.0%)	0.692	0.180, 2.668	0.593	0.349	0.052, 2.358	00.280
EZD, *n* (%)	0 (0%)	7 (35.0%)	NA	NA	NA	NA	NA	NA
DRIL, *n* (%)	4 (25.0%)	16 (80%)	12	2.484, 57.975	0.002	2.682	0.406, 17.709	0.306
HE, *n* (%)	9 (56.3%)	17 (85.0%)	4.407	0.912, 21.301	0.065	22.021	0.927, 523.131	0.056
HRF, *n* (%)	15 (93.8%)	19 (95.0%)	1.267	0.073, 21.970	0.871	33.674	0.076, 14,942.192	0.258
Number of HRF, *n* (%)								
- ≤20 (ref.)	8 (50.0%)	4 (20%)	1 (Ref.)	—	—	1 (Ref.)	—	—
- >20	7 (43.8%)	15 (75.0%)	4.286	0.958, 19.178	0.057	8.147	0.697, 95.197	0.094
LONLCs, *n* (%)	9 (56.3%)	18 (90.0%)	7	1.200, 40.828	0.031	2.142	0.297, 15.462	0.450
VMI, *n* (%)	7 (43.8%)	8 (40.0%)	0.857	0.226, 3.249	0.821	0.363	0.053, 2.465	0.300

* Adjusted by baseline CST. NA: OR was not calculated due to zero frequency in one of the cells. Fisher’s exact test showed a statistically significant difference for EZ disruption (*p* = 0.011). Abbreviations: CI, confidence interval; CST, central subfield thickness; DRIL, disorganization of the retinal inner layers; ERM, epiretinal membrane; EZD, ellipsoid zone disruption; HEs, hard exudates; HRF, hyperreflective foci; IRCs, intraretinal cysts; LONLCs, large outer-nuclear-layer cavities; OCT, optical coherence tomography; OR, odds ratio; SD, standard deviation; SRF, subretinal fluid; VMI, vitreomacular interface.

**Table 3:** OCT biomarkers as predictors for suboptimal responders.

Parameter	Good Responders (*n* = 12)	Suboptimal Responders (*n* = 24)	Univariate			Multivariate		
			Crude OR	95% CI	*p* Value	Adjusted OR *	95% CI	*p* Value
CST, Mean ± SD	315.5 ± 39.1	406.8 ± 95.6	1.019	1.003, 1.036	0.022	—	—	—
SRF, *n* (%)	1 (8.3%)	5 (20.8%)	2.895	0.299, 28.070	0.359	1.547	0.121, 19.713	0.737
IRC, *n* (%)	7 (58.3%)	22 (91.7%)	7.857	1.239, 49.834	0.029	3.516	0.491, 25.170	0.211
ERM, *n* (%)	6 (50.0%)	8 (33.3%)	0.500	0.122, 2.057	0.337	0.292	0.051, 1.664	0.165
EZD, *n* (%)	0 (0%)	7 (29.2%)	NA	NA	NA	NA	NA	NA
DRIL, *n* (%)	4 (33.3%)	16 (66.7%)	4.000	0.920, 17.396	0.065	0.908	0.142, 5.789	0.918
HE, *n* (%)	8 (66.7%)	18 (75.0%)	1.500	0.330, 6.822	0.600	1.498	0.264, 8.491	0.648
HRF, *n* (%)	12 (100%)	22 (91.7%)	NA	NA	NA	NA	NA	NA
Number of HRF, *n* (%)								
- ≤20 (ref.)	5 (41.7%)	7 (31.8%)	1 (Ref.)	—	—	1 (Ref.)	—	—
- >20	7 (58.3%)	15 (68.2%)	1.531	0.357, 6.569	0.567	1.124	0.214, 5.907	0.890
LONLCs, *n* (%)	6 (50.0%)	21 (87.5%)	7.000	1.336, 36.686	0.021	3.041	0.510, 18.139	0.222
VMI, *n* (%)	6 (50.0%)	9 (37.5%)	0.600	0.148, 2.436	0.475	4.412	0.404, 48.211	0.224

* Adjusted by baseline CST. Good responders: Based on the criteria established by the Diabetic Retinopathy Clinical Research Network (DRCR.net) Protocol T, anatomical remission was defined as a central subfield thickness (CST) of <320 µm for men and <305 µm for women using Heidelberg Spectralis OCT; Suboptimal Responders: Persistence or recurrence; NA: OR was not calculated due to zero frequency in one of the cells. Fisher’s exact test showed no statistically significant difference for EZ disruption (*p* = 0.070) and HRF (*p* = 0.543). Abbreviations: CI, confidence interval; CST, central subfield thickness; DRIL, disorganization of the retinal inner layers; ERM, epiretinal membrane; EZD, ellipsoid zone disruption; HEs, hard exudates; HRF, hyperreflective foci; IRCs, intraretinal cysts; LONLCs, large outer-nuclear-layer cavities; OCT, optical coherence tomography; OR, odds ratio; SD, standard deviation; SRF, subretinal fluid; VMI, vitreomacular interface.

**Table 4:** General linear model assessing baseline OCT biomarkers as independent predictors for the magnitude of CST reduction, adjusted for baseline CST.

Parameter	Unstandardized Coeff. (*B*)	95% CI for *B*	Standardized Coeff. (β)	*p* Value
SRF	28.927	−10.010, 67.865	0.135	0.140
IRC	9.423	−30.043, 48.888	0.047	0.630
ERM	−8.720	−38.714, 21.274	−0.053	0.558
EZD	25.916	−23.962, 75.794	0.129	0.298
DRIL	10.463	−27.298, 48.224	0.065	0.577
HE	45.984	17.503, 74.465	0.259	0.002
HRF	−8.206	−73.160, 56.748	−0.024	0.799
Number of HRF				
- ≤20 (ref.)	—	—	—	—
- >20	24.419	−3.753, 52.591	0.160	0.087
LONLCs	3.629	−33.407, 40.666	0.020	0.843
VMI	−10.708	−40.513, 19.096	−0.066	0.470

## Data Availability

All relevant data have been included in this study. The data generated from this study are available from the corresponding author upon reasonable request.

## Supplementary Materials

The following supporting information can be downloaded at https://www.mdpi.com/article/10.3390/jcm15166356/s1. Table S1: Baseline biomarker status, stratified by baseline clinical parameters; Table S2: Biomarker changes after study, stratified by different outcome groups; Figure S1: Average BCVA in all patients by treatment duration; Figure S2: Average IOP in all patients by treatment duration.

## Abbreviations

The following abbreviations are used in this manuscript:

Ang-2	Angiopoietin-2
BCVA	Best-corrected visual acuity
CI	Confidence interval
CI-DME	Center-involved diabetic macular edema
CST	Central subfield thickness
ΔCST	Change in central subfield thickness
DME	Diabetic macular edema
DRIL	Disorganization of the retinal inner layers
ERM	Epiretinal membrane
EZ	Ellipsoid zone
EZD	Ellipsoid zone disruption
GLM	General linear model
HEs	Hard exudates
HRF	Hyperreflective foci
IOP	Intraocular pressure
IRCs	Intraretinal cysts
IVF	Intravitreal faricimab
LogMAR	Logarithm of the Minimum Angle of Resolution
LONLCs	Large outer-nuclear-layer cavities
NPDR	Non-proliferative diabetic retinopathy
OCT	Optical coherence tomography
PDR	Proliferative diabetic retinopathy
PRN	Pro re nata
RPE	Retinal pigment epithelium
SD	Standard deviation
SD-OCT	Spectral-domain optical coherence tomography
SRF	Subretinal fluid
T&E	Treat-and-extend
VEGF	Vascular endothelial growth factor
VMI	Vitreomacular interface
