# Supplementary material for: Beyond Central Subfield Thickness: Early Multi-Slice Optical Coherence Tomography Structural Response After Faricimab Injection in Real-World Diabetic Macular Edema
Source: J Clin Med. 2026 Aug 17;15(16):6356. doi: 10.3390/jcm15166356 (PMC13514210; doi:10.3390/jcm15166356)
Supplement: Supplementary file 1 [file jcm-15-06356-s001.zip › Figure S1.pdf]

Figure S1. Average BCVA in all patients by treatment duration.

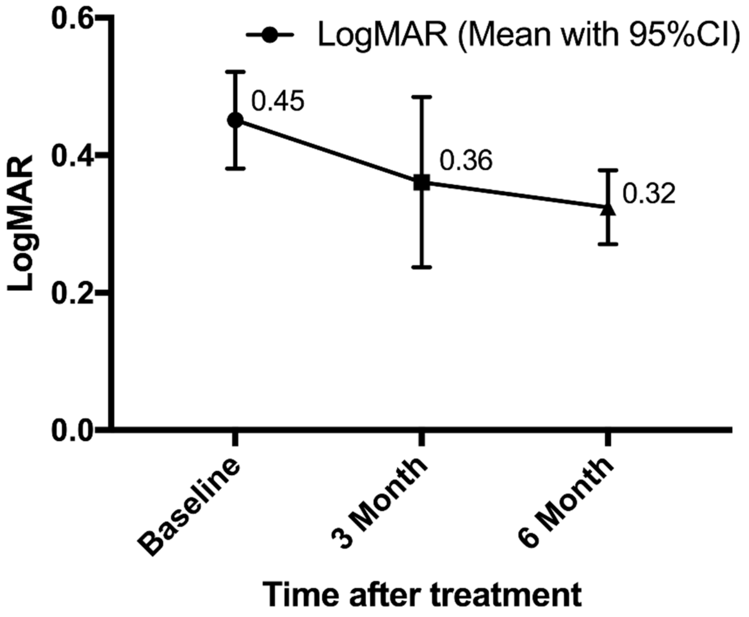

Time	Baseline	3 Month	6 Month
LogMAR (Mean ± SD)	0.45 ± 0.42	0.36 ± 0.31	0.32 ± 0.32
95% CI	0.30 - 0.59	0.24 - 0.48	0.22 - 0.43

Figure S1. Mean best-corrected visual acuity (BCVA) at baseline, 3-month, and 6-month follow-up visits. Error bars represent 95% confidence intervals. Abbreviations: BCVA, best-corrected visual acuity; CI, confidence interval; LogMAR, Logarithm of the Minimum Angle of Resolution; SD, standard deviation.
