# Supplementary material for: Beyond Central Subfield Thickness: Early Multi-Slice Optical Coherence Tomography Structural Response After Faricimab Injection in Real-World Diabetic Macular Edema
Source: J Clin Med. 2026 Aug 17;15(16):6356. doi: 10.3390/jcm15166356 (PMC13514210; doi:10.3390/jcm15166356)
Supplement: Supplementary file 1 [file jcm-15-06356-s001.zip › Figure S2.pdf]

Figure S2. Average IOP in all patients by treatment duration.

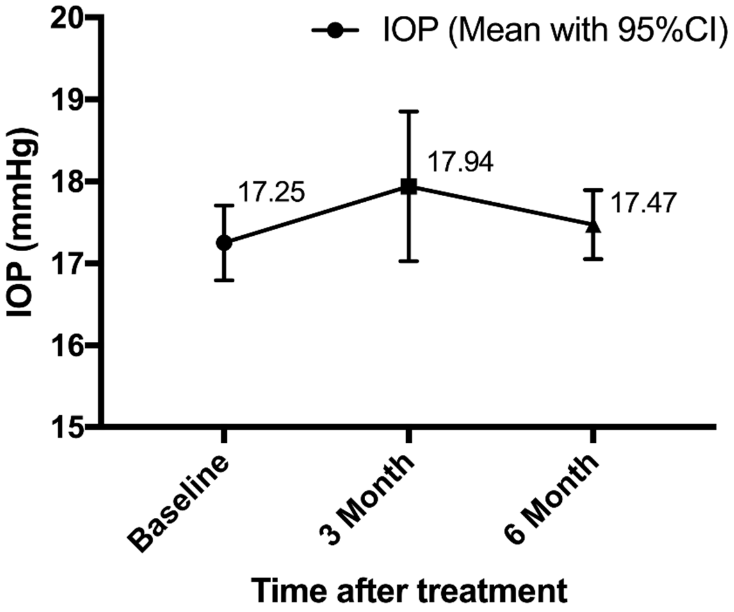

Time	Baseline	3 Month	6 Month
IOP (Mean ± SD)	17.25 ± 2.74	17.94 ± 2.62	17.47 ± 2.53
95% CI	16.3 - 18.2	17.0 - 18.9	16.6 - 18.3

Figure S2. Mean IOP measurements at baseline, 3-month, and 6-month follow-up visits. Error bars represent 95% confidence intervals. Abbreviations: CI, confidence interval; IOP, intraocular pressure; SD, standard deviation.
