# Supplementary material for: Beyond Central Subfield Thickness: Early Multi-Slice Optical Coherence Tomography Structural Response After Faricimab Injection in Real-World Diabetic Macular Edema
Source: J Clin Med. 2026 Aug 17;15(16):6356. doi: 10.3390/jcm15166356 (PMC13514210; doi:10.3390/jcm15166356)
Supplement: Supplementary file 1 [file jcm-15-06356-s001.zip › Table S1.pdf]

Table S1. Baseline biomarker status, stratified by baseline clinical parameters (eyes with positive markers).

Biomarkers (No.)	All patients (n=36)	Age			Initial BCVA (LogMAR)			Initial CST			DR Severity		
		≤65 (n= 24)	>65 (n=12)	P value	≤0.4 (n=23)	>0.4 (n=13)	P value	≤350 (n=19)	>350 (n=17)	P value	Lower severity group (n=13)	Higher severity group (n=22)	P value
SRF, n (%)	6 (16.7%)	2 (8.3%)	4 (33.3%)	.149	1 (4.3%)	5 (38.5%)	**.016**	2 (10.5%)	4 (23.5%)	.391	2 (15.4%)	4 (18.2%)	1.00
IRC, n (%)	29 (80.6%)	20 (83.3%)	9 (75.0%)	.664	17 (73.9%)	12 (92.3%)	.382	13 (68.4%)	16 (94.1%)	.092	10 (76.9%)	19 (81.8%)	.686
ERM, n (%)	14 (38.9%)	9 (37.5%)	5 (41.6%)	1.00	9 (39.1%)	5 (38.5%)	1.00	7 (36.8%)	7 (41.2%)	1.00	3 (23.1%)	11 (50.0%)	.162
EZD, n (%)	7 (19.4%)	2 (8.3%)	5 (41.7%)	**.029**	1 (4.3%)	6 (46.2%)	**.005**	0 (0%)	7 (41.2%)	**.002**	1 (7.7%)	6 (27.3%)	.382
DRIL, n (%)	20 (55.6%)	12 (50.0%)	8 (66.7%)	.481	12 (52.2%)	8 (61.5%)	.731	5 (26.3%)	15 (88.2%)	**<0.001**	6 (46.2%)	14 (59.1%)	.493
HE, n (%)	26 (72.2%)	17 (70.8%)	9 (75.0%)	1.00	15 (65.2%)	11 (84.6%)	.270	14 (73.7%)	12 (70.6%)	1.00	10 (76.9%)	16 (68.2%)	.716
HRF, n (%)	34 (94.4%)	22 (91.7%)	12 (100%)	.543	21 (91.3%)	13 (100%)	.525	19 (100%)	15 (88.2%)	.216	12 (92.3%)	22 (95.5%)	1.00
Number of HRF, n (%)				.165			.200			.374			.682
- 2-10	6 (17.6%)	3 (13.6%)	3 (25.0%)	—	5 (23.8%)	1 (7.7%)	—	5 (26.3%)	1 (6.7%)	—	2 (16.7%)	4 (19.0%)	—
- 10-20	6 (17.6%)	6 (27.3%)	0 (0%)	—	5 (23.8%)	1 (7.7%)	—	3 (15.8%)	3 (20.0%)	—	1 (8.3%)	4 (19.0%)	—
- >20	22 (64.7%)	13 (59.1%)	9 (75.0%)	—	11 (52.4%)	11 (84.6%)	—	11 (57.9%)	11 (73.3%)	—	9 (75.0%)	13 (61.9%)	—
HRF location, n (%)				.781			.765			.742			.781
- All layer	30 (88.2%)	19 (86.4%)	11 (91.7%)	—	18 (85.7%)	12 (92.3%)	—	16 (84.2%)	14 (93.3%)	—	11 (91.7%)	19 (85.7%)	—
- OPL-ONL	2 (5.9%)	1 (4.5%)	1 (8.3%)	—	1 (4.8%)	1 (7.7%)	—	2 (10.5%)	0 (0%)	—	1 (8.3%)	1 (4.8%)	—
- ILM-INL	2 (5.9%)	2 (9.1%)	0 (0%)	—	2 (9.5%)	0 (0%)	—	1 (5.3%)	1 (6.7%)	—	0 (0%)	2 (9.5%)	—
LONLC, n (%)	27 (75.0%)	19 (79.2%)	8 (66.7%)	.443	17 (73.9%)	10 (76.9%)	1.00	11 (57.9%)	16 (94.1%)	**.020**	9 (69.2%)	18 (77.3%)	.693
VMI, n (%)	15 (41.7%)	10 (41.7%)	5 (41.7%)	1.00	10 (43.5%)	5 (38.5%)	1.00	7 (36.8%)	6 (47.1%)	.736	3 (23.1%)	11 (50.0%)	.162

Abbreviations: BCVA, best-corrected visual acuity; CST, central subfield thickness; DR, diabetic retinopathy; DRIL, disorganization of the retinal inner layers; ERM, epiretinal membrane; EZD, ellipsoid zone disruption; HE, hard exudates; HRF, hyperreflective foci; ILM-INL, internal limiting membrane–inner nuclear layer; IRC, intraretinal cysts; LONLC, large outer nuclear layer cavities; LogMAR, logarithm of the minimum angle of resolution; OPL-ONL, outer plexiform layer–outer nuclear layer; SRF, subretinal fluid; VMI, vitreomacular interface.
