# Supplementary material for: Beyond Central Subfield Thickness: Early Multi-Slice Optical Coherence Tomography Structural Response After Faricimab Injection in Real-World Diabetic Macular Edema
Source: J Clin Med. 2026 Aug 17;15(16):6356. doi: 10.3390/jcm15166356 (PMC13514210; doi:10.3390/jcm15166356)
Supplement: Supplementary file 1 [file jcm-15-06356-s001.zip › Table S2.pdf]

Table S2. Biomarker changes after study, stratified by different outcome groups.

Biomarkers (No.)	All patients			Final CST response						Final anatomical responders						Final BCVA response					
				Final CST improved <50 µm (n=16)			Final CST improved ≥ 50µm (n=20)			Good responders (n=12)			Suboptimal Responders (n=24)			Final BCVA not improved (ΔLogMAR ≥ 0) (n=18)			Final BCVA improved (ΔLogMAR < 0) (n=18)		
	Baseline	End	*P* value	Baseline	End	*P* value	Baseline	End	*P* value	Baseline	End	*P* value	Baseline	End	*P* value	Baseline	End	*P* value	Baseline	End	*P* value
SRF, n (%)	6 (16.7%)	1 (2.8%)	.063	1 (6.3%)	0 (0%)	NA	5 (25.0%)	1 (5.0%)	.125	—	—	—	5 (20.8%)	1 (4.2%)	.125	2 (11.1%)	0 (0%)	NA	4 (22.2%)	1 (5.6%)	.250
IRC, n (%)	29 (80.6%)	22 (61.1%)	**.016**	10 (62.5%)	8 (50.0%)	.500	19 (95.0%)	14 (70.0%)	.063	—	—	—	22 (91.7%)	22 (91.7%)	1.00	15 (83.3%)	12 (66.7%)	.250	14 (77.8%)	10 (55.6%)	.125
ERM, n (%)	14 (38.9%)	14 (38.9%)	1.00	7 (43.8%)	7 (43.8%)	1.00	7 (35.0%)	7 (35.0%)	1.00	6 (50.0%)	6 (50.0%)	1.00	8 (33.3%)	8 (33.3%)	1.00	8 (44.4%)	8 (44.4%)	1.00	6 (33.3%)	6 (33.3%)	1.00
EZD, n (%)	7 (19.4%)	9 (25.0%)	.500	0 (0%)	2 (12.5%)	NA	7 (35.0%)	7 (35.0%)	1.00	0 (0%)	1 (8.3%)	NA	7 (29.2%)	8 (33.3%)	1.00	3 (16.7%)	4 (22.2%)	1.00	4 (22.2%)	5 (27.8%)	1.00
DRIL, n (%)	20 (55.6%)	12 (33.3%)	**.021**	4 (25.0%)	4 (25.0%)	1.00	16 (80.0%)	8 (40.0%)	**.008**	4 (33.3%)	2 (16.7%)	.625	16 (66.7%)	10 (41.7%)	**.031**	10 (55.6%)	7 (38.9%)	.250	10 (55.6%)	5 (27.8%)	.125
HE, n (%)	26 (72.2%)	25 (69.4%)	1.00	9 (56.3%)	9 (56.3%)	1.00	17 (85.0%)	16 (80.0%)	1.00	8 (66.7%)	7 (58.3%)	1.00	18 (75.0%)	18 (75.0%)	1.00	12 (66.7%)	11 (61.1%)	1.00	14 (77.8%)	14 (77.8%)	1.00
HRF, n (%)	34 (94.4%)	35 (97.2%)	1.00	15 (93.8%)	15 (93.8%)	1.00	19 (95.0%)	20 (100%)	NA	12 (100%)	12 (100%)	NA	22 (91.7%)	23 (95.8%)	1.00	16 (88.9%)	17 (94.4%)	1.00	18 (100.0%)	18 (100.0%)	NA
Number of HRF, n (%)																					
- ≤20 (ref.)	12 (35.3%)	16 (47.1%)	—	8 (53.3%)	8 (53.3%)	—	4 (21.1%)	8 (42.1%)	—	5 (41.7%)	9 (75.0%)	—	7 (31.8%)	7 (31.8%)	—	7 (43.8%)	7 (43.8%)	—	5 (27.8%)	9 (50.0%)	—
- >20	22 (64.7%)	18 (52.9%)	.289	7 (46.7%)	7 (46.7%)	1.00	15 (78.9%)	11 (57.9%)	.219	7 (58.3%)	3 (25.0%)	.125	15 (68.2%)	15 (68.2%)	1.00	9 (56.3%)	9 (56.3%)	1.00	13 (72.2%)	9 (50.0%)	.219
LONLC, n (%)	27 (75.0%)	12 (33.3%)	**< .001**	9 (56.3%)	4 (25.0%)	.063	18 (90.0%)	8 (40.0%)	**.002**	—	—	—	21 (87.5%)	12 (50.0%)	**.004**	15 (83.3%)	7 (38.9%)	**.008**	12 (66.7%)	5 (27.8%)	**.016**
VMI, n (%)	15 (41.7%)	15 (41.7%)	1.00	7 (43.8%)	7 (43.8%)	1.00	8 (40.0%)	8 (40.0%)	1.00	6 (50.0%)	6 (50.0%)	1.00	9 (37.5%)	9 (37.5%)	1.00	9 (50.0%)	9 (50.0%)	1.00	6 (33.3%)	6 (33.3%)	1.00

Analysis by McNemar's test

NA: *P* value was not calculated due to zero frequency in one of the cells.

Good responders: Based on the criteria established by the Diabetic Retinopathy Clinical Research Network (DRCR.net) Protocol T, anatomical remission was defined as a central subfield thickness (CST) of <320 µm for men and <305 µm for women using Heidelberg Spectralis OCT.

Abbreviations: BCVA, best-corrected visual acuity; CST, central subfield thickness; DR, diabetic retinopathy; DRIL, disorganization of the retinal inner layers; ERM, epiretinal membrane; EZD, ellipsoid zone disruption; HE, hard exudates; HRF, hyperreflective foci; IRC, intraretinal cysts; LONLC, large outer nuclear layer cavities; LogMAR, logarithm of the minimum angle of resolution; SRF, subretinal fluid; VMI, vitreomacular interface.
